# Deep-learned time-signal intensity pattern analysis using an autoencoder captures magnetic resonance perfusion heterogeneity for brain tumor differentiation

**DOI:** 10.1038/s41598-020-78485-x

**Published:** 2020-12-08

**Authors:** Ji Eun Park, Ho Sung Kim, Junkyu Lee, E.-Nae Cheong, Ilah Shin, Sung Soo Ahn, Woo Hyun Shim

**Affiliations:** 1 Department of Radiology and Research Institute of Radiology, University of Ulsan College of Medicine, Asan Medical Center, 43 Olympic-ro 88, Songpa-Gu, Seoul, 05505 Korea; 2 Department of Medical Science and Asan Medical Institute of Convergence Science and Technology, Asan Medical Center, University of Ulsan College of Medicine, 43 Olympic-ro 88, Songpa-Gu, Seoul, Korea; 3 Department of Radiology, Research Institute of Radiological Science and Center for Clinical Imaging Data Science, Yonsei University College of Medicine, Seoul, Korea

**Keywords:** Diagnostic markers, Cancer imaging, CNS cancer

## Abstract

Current image processing methods for dynamic susceptibility contrast (DSC) magnetic resonance imaging (MRI) do not capture complex dynamic information of time-signal intensity curves. We investigated whether an autoencoder-based pattern analysis of DSC MRI captured representative temporal features that improves tissue characterization and tumor diagnosis in a multicenter setting. The autoencoder was applied to the time-signal intensity curves to obtain representative temporal patterns, which were subsequently learned by a convolutional neural network. This network was trained with 216 preoperative DSC MRI acquisitions and validated using external data (n = 43) collected with different DSC acquisition protocols. The autoencoder applied to time-signal intensity curves and clustering obtained nine representative clusters of temporal patterns, which accurately identified tumor and non-tumoral tissues. The dominant clusters of temporal patterns distinguished primary central nervous system lymphoma (PCNSL) from glioblastoma (AUC 0.89) and metastasis from glioblastoma (AUC 0.95). The autoencoder captured DSC time-signal intensity patterns that improved identification of tumoral tissues and differentiation of tumor type and was generalizable across centers.

## Introduction

Dynamic susceptibility contrast (DSC) MRI is the most commonly used perfusion technique in clinical practice^1^ and in clinical trial^2, 3^ for evaluation of brain tumor vascularity. Much of the discussion of DSC MRI has focused on measurements using relative cerebral blood volume (rCBV)^4–7^, and other parameters of relative recirculation^8, 9^ and vascular permeability^10–12^. However, these techniques are limited to fully capture dynamic information that DSC MRI retains. Every voxel in tumors exhibits different series of signal intensities according to time, and analysis of stationary and one-dimensional (scalar) values do not allow for a complete characterization of dynamic perfusion information. Also, model-based calculation of rCBV and relative recirculation requires sophisticated assumption and introduces potential sources of variances^8, 13, 14^ from different pulse sequence parameters, contrast media, model assumption from different hospital setting^15^. Unsupervised learning of time-series data, on the other hand, has a great potential to learn complex time-signal intensity data in DSC MRI^16, 17^, which can provide additional information that cannot be captured with current perfusion MRI processing.

Time-signal intensity patterns can be learned by an autoencoder neural network for high-dimensional time-series data^16^. This method uses an unsupervised learning algorithm to learn a compressed representation of the input data. The autoencoder has an encoder-decoder architecture that removes unnecessary noise and generates representations from a low-dimensional latent space by stacking multiple non-linear transformations^16, 18^. This architecture enables the modeling of complex non-linear functions, unlike linear transformations in principal component analysis^19^. Also, the low-dimensional latent space in the autoencoder can encode the most important features of the input data, which can then be used in data visualization and become a representation for a supervised learning model.

We hypothesized that the autoencoder would capture temporal patterns from DSC MRI data that are not accessible with current perfusion MRI processing, whichwould yield high diagnostic values in brain tumors with clinical relevance. We investigated whether autoencoder-derived pattern analysis of DSC MRI data captured representative temporal features and augmented tumor characterization in terms of heterogeneity and across multiple centers.

## Methods

### Patients

**Figure 1:**
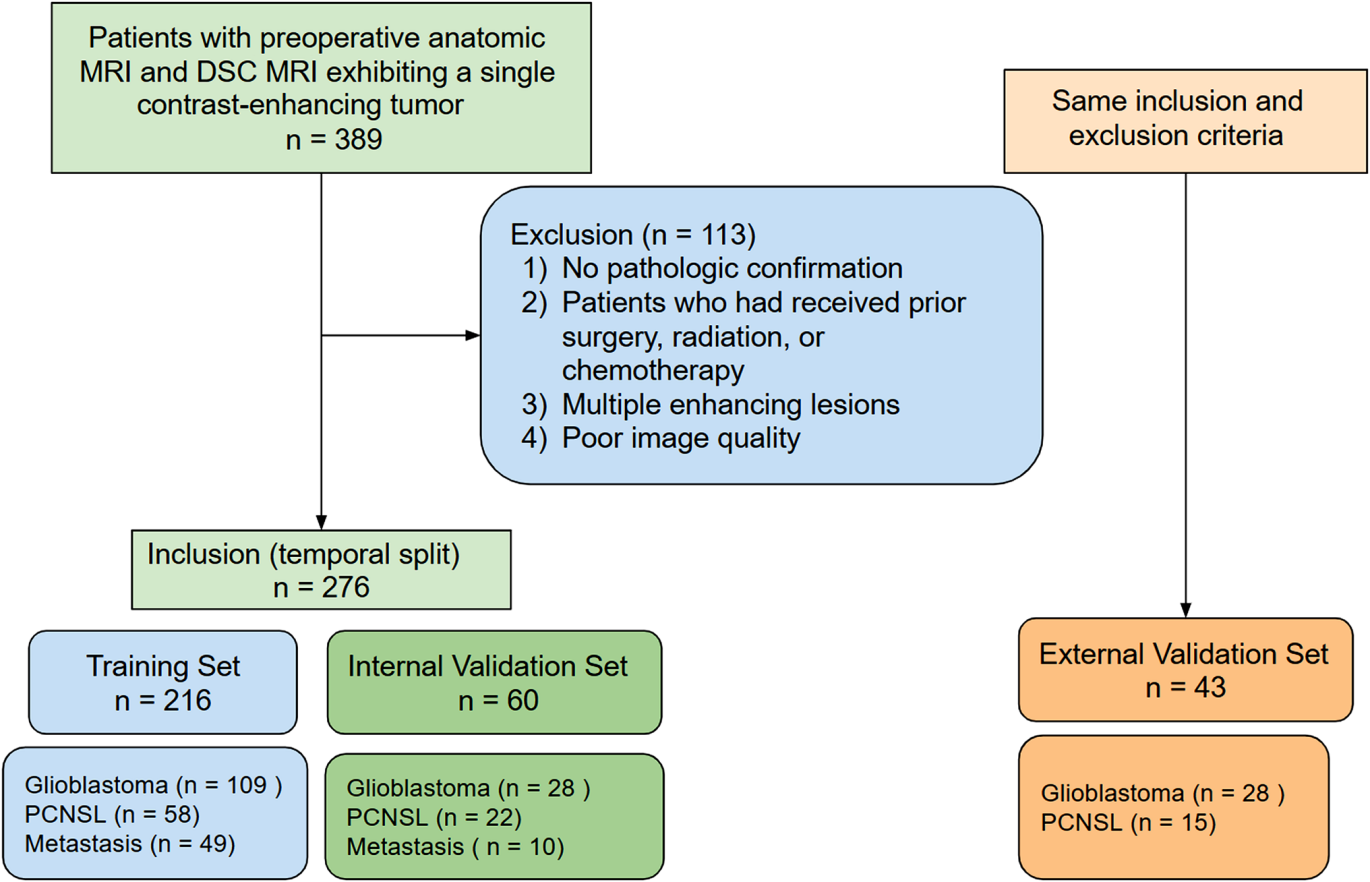
The inclusion process of the study patients.

This retrospective study was approved by the Institutional Review Board of the Asan Medical Center (approval no. 2019-0594), and all methods were carried out in accordance with the institution’s guidelines and regulations. The requirement for informed consent was formally waived by the approving committee. The electronic database of the Department of Radiology at the University of Ulsan, College of Medicine was searched and records for patients between September 2013 and March 2019 were retrospectively reviewed. In total, 389 patients were identified. The inclusion process is shown in Fig. 1. The inclusion criteria were patients with a single contrast-enhancing tumor revealed by preoperative anatomic MRI and DSC MRI. Exclusion criteria were as follows: (1) no pathologic confirmation; (2) patients who had a prior tumor treated with surgery, radiation, or chemotherapy; (3) multiple contrast-enhancing tumors; and (4) poor image quality. Twenty-three patients were excluded because of prior resection or therapy, and 30 patients were excluded because of multiple tumors. In addition, 50 patients were excluded because DSC MRI data were not available, and 10 patients were excluded because of poor image quality caused by artifacts. After applying the exclusion criteria, there were 276 remaining patients (mean age 60.4 years; male: female ratio 152: 124). Of these patients, 137 had glioblastoma, 80 had lymphoma, and 59 had brain metastasis. The autoencoder was developed using training, and internal validation set was formed based on a temporal split of the institutional data obtained in September 2013 and July 2018. The training set included 216 patients (glioblastoma:PCNSL:metastasis = 109:58:49). The internal validation set included 60 patients (glioblastoma:PCNSL:metastasis = 28:22:10).

To test the generalizability and performance of the algorithm^20^, an external validation set consisting of 43 patients from another tertiary medical center (Severance Hospital, Seoul, Korea) was included. A total of 28 patients with pathologically confirmed glioblastoma and 15 patients with PCNSL were used for external validation of the model.

### Imaging data

All MRI sessions on enrolled patients at both institutions were performed on a 3 T unit (Achieva or Ingenia; Philips Medical Systems, Best, Netherlands) equipped with a 32-channel head coil. The brain-tumor imaging protocol included the following sequences: T2-weighted, fluid-attenuated inversion recovery (FLAIR), T1-weighted, diffusion-weighted, contrast-enhanced T1-weighted (CE-T1WI), and DSC perfusion MRI. The DSC data were collected using T2*-weighted gradient echo-planar images.

The DSC MRI protocol used for the training and internal validation sets began with a preload of 0.1 mmol/kg gadoterate meglumine (Dotarem; Guerbet), was given before the dynamic bolus. Then, the dynamic bolus was administered as a standard dose of 0.1 mmol/kg gadoterate meglumine delivered at a rate of 4 mL/s by an MRI-compatible power injector (Spectris; Medrad). The bolus of contrast material was followed by a 20 mL bolus of saline, which was injected at the same rate. The pulse sequence parameters included the following: repetition time (TR)/echo time (TE), 1724/40 ms; flip angle, 35°; field of view (FOV), 240 mm; slice thickness/gap, 5/2 mm; matrix, 128 × 128; total acquisition time, 1 min and 50 s. Dynamic acquisition was performed with a temporal resolution of 1.7 s, and 60 dynamics were acquired. Each timepoint included 20 slices, covering the whole brain in the same orientation as that used for the conventional sequences.

In the external validation set, DSC images were also obtained with a preload of gadobutrol (0.1 mmol/kg Gadovist, Bayer Schering Pharma) given before the dynamic bolus. Then, a 0.1 mmol/kg dynamic bolus of gadobutrol was injected at a rate of 4 mL/s, followed by a bolus injection of saline (total 20 mL at 4 mL/s). The pulse sequence parameters included the following: TR/TE, 1600/30 ms; flip angle, 40°; FOV, 220 mm; slice thickness/gap, 5/1.8 mm; 128 × 128 matrix. The dynamic acquisition was performed with a temporal resolution of 1.6 s, and 60 dynamics were acquired.

### Labeling of brain tissue and brain tumor

The CE-T1WI and FLAIR images were co-registered to the DSC images to define brain regions. The co-registration process was performed in Statistical Parametric Mapping (SPM12) and used affine transformations with six degrees of freedom and 4th degree B-spline interpolation. The probability template matching in SPM12 was also used to segment and label brain regions^21^ into gray matter, white matter, and cerebral spinal fluid (CSF). Non-brain tissues were excluded in this step.

Tumor segmentation was performed semi-automatically by a neuroradiologist (2 years of experience in neuro-oncological imaging) using the CE-T1WI and FLAIR data. Necrosis and the contrast-enhancing solid portion were selected on the 3D CE-T1WI. The FLAIR images were used to select edema/non-enhancing tumor portions using a segmentation threshold and region-growing segmentation algorithm that was implemented using MITK software (www.mitk.org; German Cancer Research Center, Heidelberg, Germany)^22^. All segmented images were checked by an expert radiologist (6 years of experience in neuro-oncological imaging). For glioblastoma cases, contrast-enhancing tumors, non-enhancing tumors, and necrosis were all segmented. For lymphoma and metastasis cases, contrast-enhancing tumors were segmented. In total, there were nine pre-defined different tissue types in the co-registered DSC images: three types of contrast-enhancing tumors (glioblastoma, lymphoma, and metastasis), non-enhancing tumors, edema, gray matter, white matter, CSF, and necrosis.

### Calculation of autoencoder representations

DSC time-signal intensity curves were calculated and the scale was normalized based on the median value of white matter for each brain. Then, outliers were removed from the time-series data by excluding data that were ± 1 standard deviation outside the mean signal. After this process, DSC time-signal intensity curves included for each patient were approximately 4.6 × 10^4^ time-signal intensity curves (mean 46,342, standard deviation, 12,765).

Representations from high-dimensional DSC time-signal intensity data were trained using a single-layer sparse autoencoder.

Each autoencoder consisted of two parts: the encoder and the decoder (Supplementary Fig. S1). Each time-signal intensity curve of 60 time points formed a 60-element input vector. The encoder was a one-dimensional convolutional layer using a rectified linear unit (ReLu) and a one-dimensional max pooling layer. The kernel size of the convolution layer was 2 and the filter size 32; the output size of the convolution layer was kept the same by applying the padding parameter as ‘same’. A dropout layer with 0.7 probability was introduced after the batch-normalization layer to reduce overfitting. The number of latent spaces was set to five. The decoder reconstructed data with the same size (60 time points) as the input data through two fully-connected layers. Mean squared error was used to calculate the loss between the output and input signal. The learning rate was 1 × 10^–5^ and the batch size for training was set to 10,000.

The autoencoder-derived representations captured the highest variance from DSC time-signal intensity data and all subjects under the constraint that variance was uncorrelated with each representation. These representations thus reflected various aspects of the DSC time-signal intensity curve patterns including baseline signal, depth of signal decrease, slopes of signal decrease and recovery, and percentage recovery. We trained the number of representations that accounted for more than 99% of the overall variance in the perfusion signal across individuals.

Then, k-means clustering was applied to the autoencoder-derived representations. A total of nine clusters were identified from the autoencoder representations, and the autoencoder representations in the latent space were decoded into decoded time-signal intensity curves representing temporal patterns. The nine clusters of decoded time-signal intensity curves (temporal patterns) were used to characterize the nine pre-defined tissue types, which consisted of three types of contrast-enhancing tumors, non-enhancing tumors, edema, gray matter, white matter, CSF, and necrosis. For this, information on the anatomic position using an x–y-z coordinate system was obtained from the decoded time-signal intensity curves and subsequently used for anatomic mapping. The nine clusters of temporal patterns were mapped onto anatomic images for visualization and to distinguish tumoral regions from normal-appearing brain tissue.

### Supervised learning using decoded time-signal intensity curves in brain tumor

Whether the decoded time-signal intensity patterns provided clinically relevant information for brain tumor characterization and differential diagnosis in terms of intratumoral perfusion heterogeneity was investigated. For this analysis, a deep convolutional neural network (CNN) was used to learn the representative time-signal intensity patterns characterizing specific pathologic tumor types. Because there was no voxel-based confirmation of tumor and non-tumor tissue, it was assumed that two dominant patterns exhibiting the highest proportions within the contrast-enhancing lesion represented specific pathologic tumor types. Within the CEL, the proportion of each cluster was calculated. Among them, two clusters with the highest proportions were selected as ‘dominant clusters’ and the decoded time-signal intensity curves from the two dominant clusters were subsequently used as input for CNN. Thus, the input for CNN was all 1 × 60 input vectors obtained from the decoded time-signal intensity curves from the two dominant clusters. A CNN was designed with 3 convolutional blocks, 2 fully connected layers, and softmax layer for classification. The input data size was 60-element input vector from 60 time points of each time-signal intensity curve. The convolutional blocks consisted of one-dimensional convolutional layer combined by a max-pooling operation, batch-normalization, and a drop-out layer (Supplementary Fig. S2). The kernel size of convolution layer was 2 and the number of feature maps was 32, and the output size of convolution layer was kept same by applying padding parameter as ‘same’. Max pooling layer down-sampled the representations by 50% for each repetition and batch-normalization was introduced, followed by drop-out layer with 0.7 of probability. Two fully-connected layers using ReLU were applied to make the same size representatives with the number of classes and then softmax layer was applied to convert these representative values as probabilities. Softmax-cross-entropy cost function was used to calculate the loss between labels and predictions. The learning rate was 1 × 10^–8^ and the batch size for training was set to 1,000. The deep-learning architecture was implemented by using the Tensorflow1.14.

### Statistical analysis

Student’s t-tests and the Chi-square tests were used to assess differences in demographic data and the prevalence of each classification category between the training and validation sets.

#### Tumor differentiation

To test diagnostic performance, the area under the curve (AUC), from receiver operating characteristic curve analysis, was calculated. The optimal thresholds of the AUCs were determined by maximizing the sum of the sensitivity and specificity values calculated for differentiation of brain tumors. To differentiate PCNSL from glioblastoma and brain metastasis from glioblastoma, the diagnostic performance was calculated with glioblastoma as a reference diagnosis. The accuracy, sensitivity, and specificity of correctly diagnosing glioblastoma from other tumors were defined as

$$Accuracy=\frac{\mathrm{TP}+\mathrm{TN}}{\mathrm{TP}+\mathrm{TN}+\mathrm{FP}+\mathrm{FN}}, Sensitivity=\frac{\mathrm{TP}}{\mathrm{TP}+\mathrm{FN}}, Specificity=\frac{\mathrm{TN}}{\mathrm{TN}+\mathrm{FP}}$$

where TP is a true positive, TN a true negative, FP a false positive, and FN a false negative.

#### Comparison to conventional approach

The diagnostic performance of the autoencoder analysis of DSC time-signal intensity patterns was compared with that of the conventional approach using mean rCBV.

*P* values less than 0.05 were considered to indicate significant differences. Statistical analyses were performed using R statistical software (version 3.6.3, R Core Team, Vienna, Austria).

## Results

Patient baseline demographics and clinical characteristics are summarized in Table 1. The training set included 216 patients (109 glioblastoma, 58 PCNSL, and 49 metastasis), and the internal validation set had 60 patients (28 glioblastoma, 22 PCNSL, and 10 metastasis). The external validation set had 43 patients (28 glioblastomas and 15 PCNSLs). There were no significant differences in sex across the sets or in sex among patients in either the training or validation sets.

**Table 1:** Patient baseline demographic and clinical characteristics.

Pathology	Training set			*P value*	Internal validation set			*P value*	External validation set		*P value*
	Glioblastoma	PCNSL	Metastasis		Glioblastoma	PCNSL	Metastasis		Glioblastoma	PCNSL	
Patients (N)	109	58	49		28	22	10		28	15	
Number of females (N)	42	33	20	0.52	11	13	5	0.56	13	7	0.53
Age (years old)	58.1 ± 13.5	62.3 ± 11.7	61.5 ± 12.0	0.29	57.58 ± 7.96	62.7 ± 11.9	61.0 ± 11.3	0.38	57.8 ± 10.5	65.3 ± 10.9	0.06

*P* values apply to the differences among the three groups. Age is expressed as mean ± standard deviation.

*PCNSL* primary central nervous system lymphoma.

### Encoded representations and clusters of temporal patterns for tissue characterization

**Figure 2:**
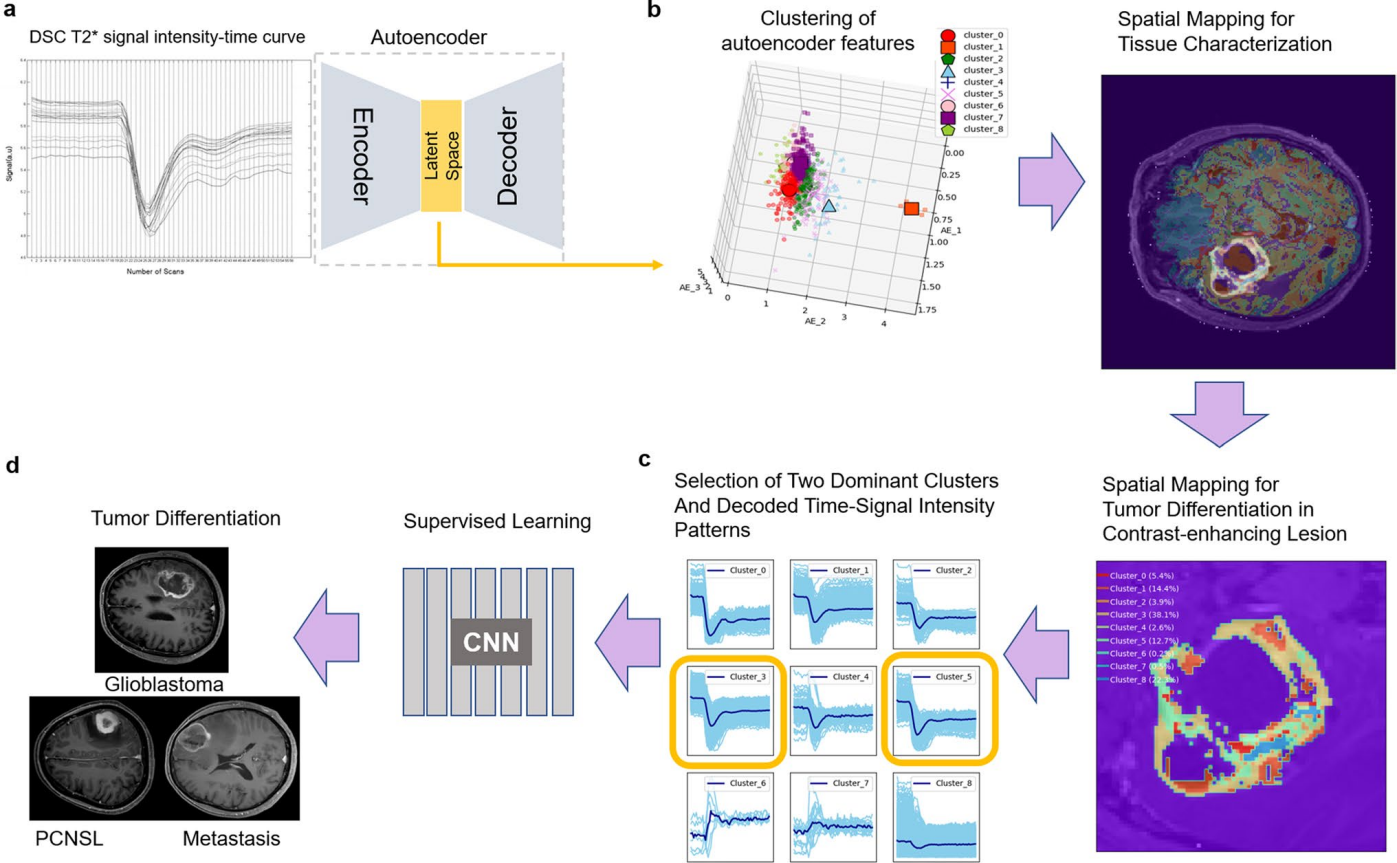
Extracting autoencoder features from dynamic susceptibility contrast (DSC) imaging. (**a**) DSC time-signal intensity curves were reduced using an autoencoder. (**b**) The autoencoder features were clustered into nine representative tissue types including contrast-enhancing lesions (CEL) (e.g., glioblastoma, lymphoma, and metastasis), non-enhancing tumor portions, edema, necrosis, gray matter, white matter, and cerebral spinal fluid (CSF). (**c**) The clusters were spatially mapped within the CEL and decoded time-signal intensity curves were clustered into nine different clusters. (**d**) A convolutional neural network (CNN) was trained with two dominant clusters of decoded time-signal intensity curves and tested for tumor differentiation. The figure was drawn using Matlab (version R2017b, Mathworks, Natick, MA) and extra permission for drawing figures was not required.

The extraction of autoencoder-derived representations, cluster visualization, and decoded time-signal intensity patterns are shown in Fig. 2.

The pre-defined tissue types corresponding to each pattern are shown in Table 2. In normally appearing brain tissue, the dominant clusters were cluster 6 (mean ± standard deviation, 24.1 ± 0.4%) in gray matter and cluster 4 (34.4 ± 3.8%) in white matter. The dominant clusters in CSF and necrosis were different, although they were bright, exhibiting high T2 signal intensity with high baseline. The dominant cluster in CSF was cluster 5 (21.6 ± 4.7%), wheres in necrosis it was cluster 1 (76.8 ± 1.0%). Using the autoencoder-derived representations, the differentiation of CEL tumors from other tissue types was 91.1% accurate in the training set and 94.6% accurate in the internal validation set.

**Table 2:** Brain tissue characterization based on frequency of patterns.

Dominant pattern		Pattern 0	Pattern 1	Pattern 2	Pattern 3	Pattern 4	Pattern 5	Pattern 6	Pattern 7	Pattern 8
CEL in glioblastoma	5 and 3	13.5 ± 1.3 (0–57.2)	0.3 ± 0.1 (0 -44.9)	14.3 ± 0.1 (0–45.7)	26.7 ± 0.2 (0 -85.0)	4.6 ± 0.0 (0–18.1)	**32.6** ± 0.1 (0–49.1)	1.4 ± 0.0 (0–41.4)	2.3 ± 0.0 (0–26.9)	4.3 ± 0.2 (0–99.2)
CEL in PCNSL	5 and 3	15.0 ± 0.2 (0–54.3)	0.1 ± 0.1 (0–59.1)	6.6 ± 0.2 (0–100)	27.7 ± 0.4 (0–100)	5.0 ± 0.1 (0–90)	**37.0** ± 0.4 (0–100)	1.9 ± 0.1 (0–41.1)	1.5 ± 0.1 (0–73.3)	5.2 ± 0.2 (0–100)
CEL in metastasis	8 and 5	13.7 ± 0.2 (0–86.9)	0 ± 0 (0–0.2)	6.3 ± 0.2 (0–97.3)	6.5 ± 0.2 (0–99.7)	9.3 ± 0.2 (0–89.2)	16.9 ± 0.3 (0–100)	4.4 ± 0.1 (0–51.7)	2.3 ± 0.1 (0–41.0)	**40.6** ± 0.4 (0–100)
Edema/Non-enhancing tumor	5 and 3	2.1 ± 0.7 (1.4–2.8)	0 ± 0 (0–0)	13.1 ± 0.6 (12.5–13.7)	27.4 ± 5.8 (21.6–33.3)	2.5 ± 1.1 (1.4–3.7)	**48.5** ± 2.8 (45.7–51.3)	0.5 ± 0.1 (0.4–0.7)	4.9 ± 1.4 (3.5–6.2)	1.0 ± 0.4 (0.5–1.4)
Gray matter	6 and 7	14.9 ± 2.8 (11.4–18.3)	0 ± 0 (0–0)	15.2 ± 1.0 (13.9–16.3)	0 ± 0 (0–0)	24.0 ± 2.9 (20.2–27.1)	0.2 ± 0.2 (0.1–0.4)	**24.1** ± 0.4 (23.6–24.7)	15.6 ± 5.2 (9.7–22.4)	6.0 ± 1.9 (3.5–8.1)
White matter	4 and 7	3.2 ± 1.5 (1.3–5.0)	0 ± 0 (0–0)	14 ± 1.9 (12.0–16.6)	0 ± 0 (0–0)	**34.4** ± 3.8 (29.7–39.1)	0 ± 0 (0–0)	19.6 ± 0.1 (19.4–19.7)	28.7 ± 3.7 (24.0–33.1)	0.1 ± 0.1 (0–0.2)
CSF	5 and 8	13.3 ± 2.0 (10.9–15.7)	0 ± 0 (0–0)	13.7 ± 1.5 (12.5–15.9)	7.1 ± 1.2 (6.1–8.8)	10.0 ± 1.5 (8.9–12.1)	**21.6** ± 4.7 (18.1–28.3)	8.9 ± 0.8 (7.9–9.9)	7.1 ± 1.5 (5.6–9.2)	18.3 ± 3.8 (13.2–22.0)
Necrosis	1	0 ± 0 (0–0)	**76.8** ± 1.0 (75.7–77.8)	1.6 ± 1.5 (0–3.1)	15.2 ± 6.0 (9.2–21.3)	1.1 ± 1.0 (0.1–2.1)	0.6 ± 0.1 (0.5–0.7)	1.5 ± 1.5 (0–3.0)	1.7 ± 1.5 (0.2–3.2)	1.5 ± 1.5 (0–2.9)

The numbers in the cells are percentages with mean ± standard deviation and the numbers in the parenthesis are minimum to maximum. Necrosis is defined in glioblastoma and metastasis.

*CEL* contrast-enhancing lesion. *PCNSL* primary central nervous system lymphoma.

**Figure 3:**
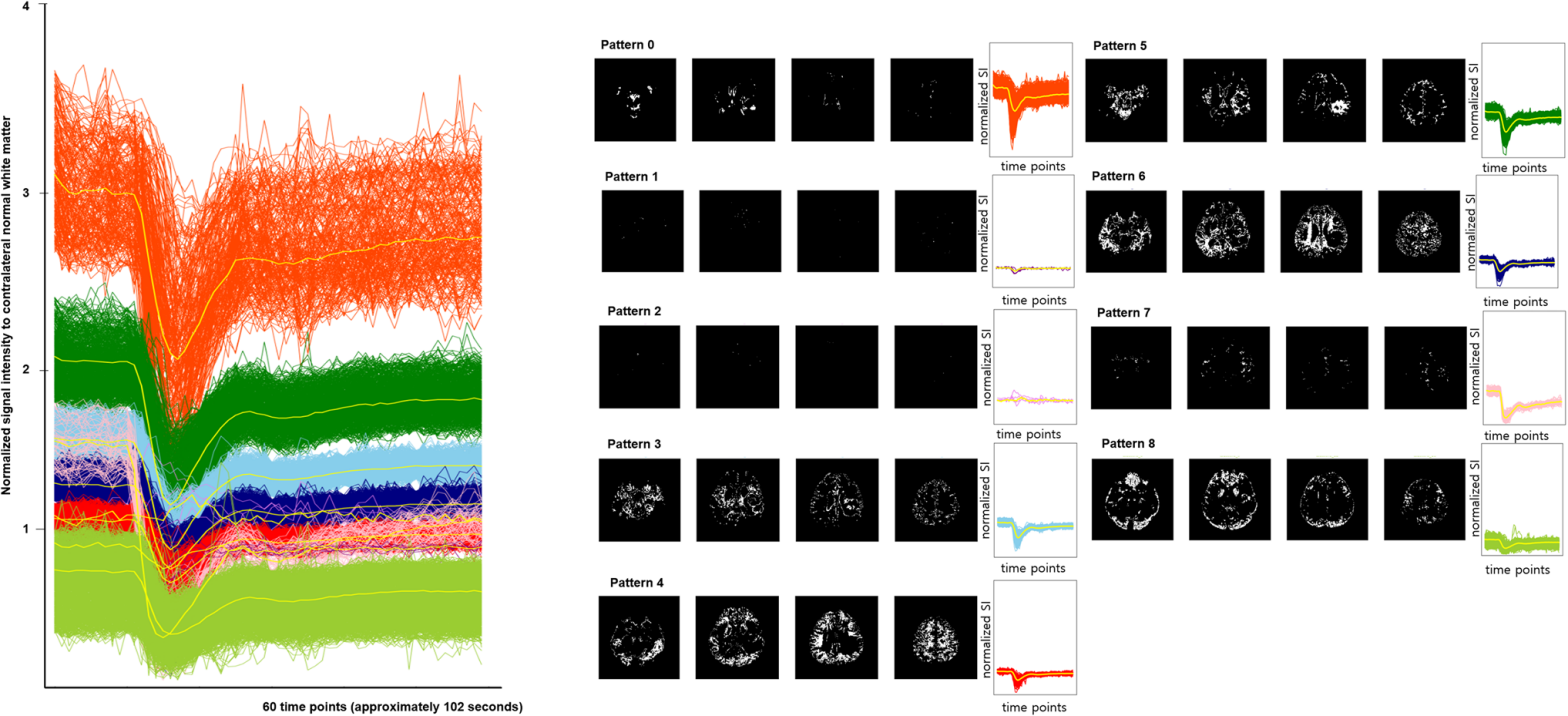
A demonstration of spatial mapping of tissue type clusters and decoded time-signal intensity curves from a glioblastoma patient. The graph on the right shows the nine representative tissue types that were clustered using autoencoder features and decoded time-signal intensity curves. The x-axis is time points (a total of 60 time points, 1.7 s for each time point) and the y-axis is the signal intensity normalized to the contralateral white matter. The resulting nine clusters differed in terms of baseline signal, depth of signal decrease, slopes of signal decrease and recovery, and relative recovery. The yellow lines show the mean time-signal intensity curves of each representative curve. The figure was drawn using Matlab (version R2017b, Mathworks, Natick, MA) and extra permission for drawing figures was not required.

Figure 3 shows the nine clustered representative temporal patterns and how the patterns were spatially mapped in the representative patients. The nine clusters of temporal patterns differed in terms of baseline signal, depth of signal decrease, slope of signal decrease and recovery, and percentage recovery. For example, clusters 4 and cluster 6 represented normal brain tissue with intermediate baseline signal intensity and a small depth of signal decrease.

### Dominant clusters of temporal patterns in brain tumor

**Figure 4:**
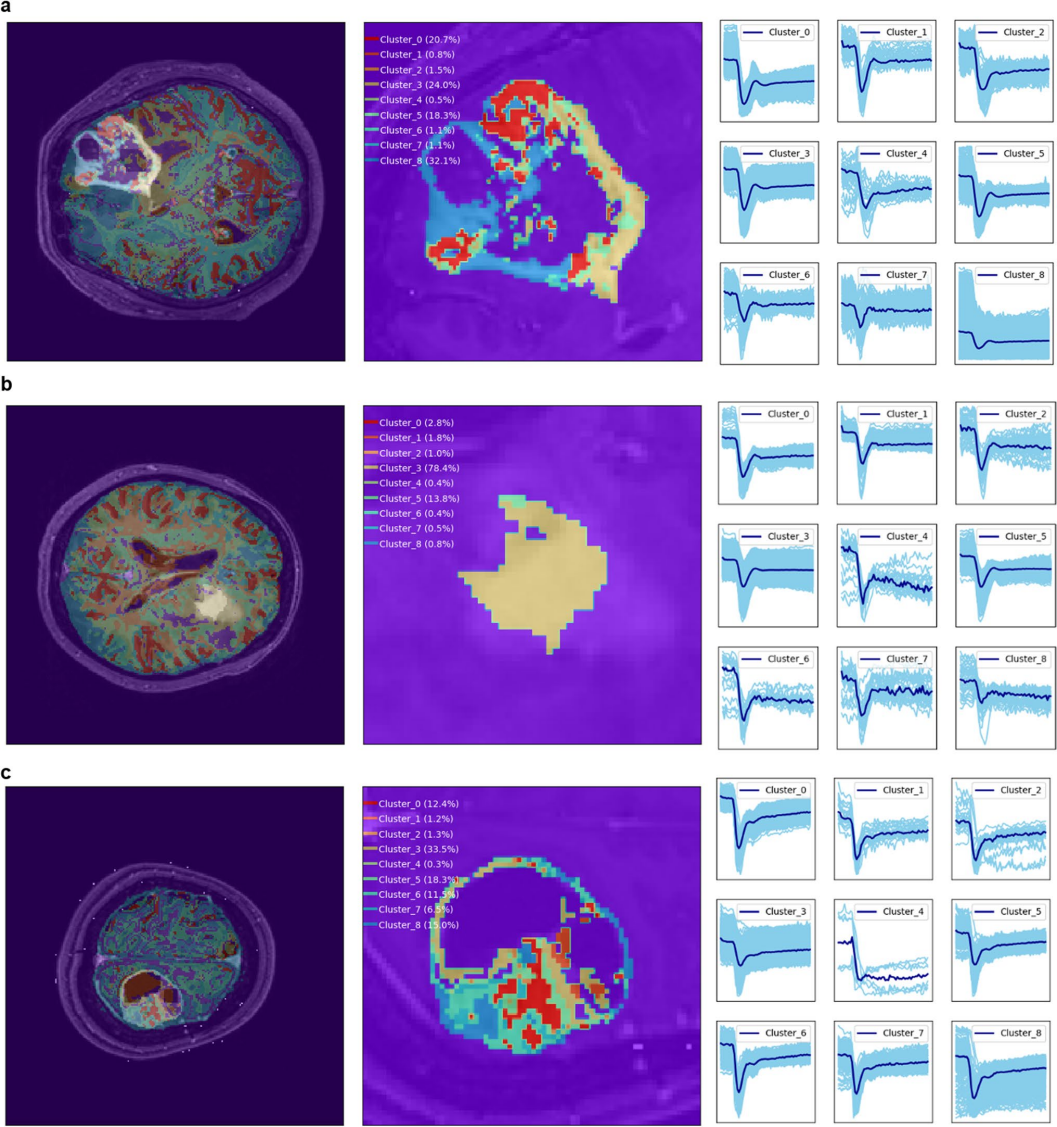
Spatial distribution of clusters of decoded time-signal intensity curves in brain tumor. (**a**) Patterns were heterogeneously distributed in glioblastoma. Dominant clusters of time-signal intensity curves (from cluster 5 and 3) showed an acute signal decrease slope and small relative recovery. (**b**) The distribution of clusters was less heterogeneous in primary central nervous system lymphoma (PCNSL) than in glioblastoma. Dominant clusters (from cluster 5 and 3) showed a less acute signal decrease slope and high relative recovery. (**c**) The distribution of clusters was also heterogeneous in metastasis. Dominant clusters (from cluster 8) showed a large depth of signal decrease and extremely low signal recovery. The figure was drawn using Matlab (version R2017b, Mathworks, Natick, MA) and extra permission for drawing figures was not required.

The dominant clusters of temporal patterns for glioblastoma were cluster 5 (32.6%) and cluster 3 (26.7%), as shown in Fig. 4a. These patterns had an intermediate baseline, acute slope of signal decrease, and a large depth of signal decrease. The relative recovery was small, and the pattern distribution was heterogeneous.

The dominant clusters for PCNSL were the same as for glioblastoma, namely cluster 5 (37.0%) and cluster 3 (27.7%). However, the frequency of cluster 3 was slightly higher in PCNSL than in glioblastoma. Figure 4b shows the dominant clusters of temporal patterns for PCNSL, which had an intermediate baseline, less acute signal decrease slope, and small depth of signal decrease. The relative recovery was high. The cluster distribution was more homogeneous in PCNSL than in glioblastoma.

The dominant clusters for metastasis were cluster 8 (40.6%) and cluster 5 (16.9%), and the corresponding temporal patterns are shown in Fig. 4c. Metastasis was characterized by a high baseline and had the most acute signal decrease slope. It also showed a large depth of signal decrease, and the relative recovery was low. Compared with PCNSL, the cluster distribution for metastasis was heterogeneous.

### Tumor differentiation and external validation

Table 3 summarizes the diagnostic performance results. Using the dominant clusters of time-signal intensity curves, the CNN distinguished PCNSL from glioblastoma in the training set with an AUC of 0.921 (95% confidence interval [CI] 0.860–0.951), sensitivity of 93.6%, and specificity of 81.0%. On the internal validation set, the CNN distinguished PCNSL from glioblastoma with an AUC of 0.93 (95% CI 0.821–0.983), sensitivity of 85.7%, and specificity of 90.9%. External validation of the CNN gave an AUC of 0.89 (95% CI 0.75–0.97), sensitivity of 95.2%, and specificity of 76.5%.

**Table 3:** Diagnostic performances of autoencoder-derived pattern analysis and conventional rCBV approach.

Parameter	AUC value (95% CI)	Cutoff	Sensitivity (%)	Specificity (%)
**Differentiation of glioblastoma from PCNSL**				
Internal validation set				
Autoencoder-derived pattern analysis	0.93 (0.82–0.98)	NA	85.7	90.1
rCBV	0.79 (0.65–0.90)	3.5	57.1	95.0
External validation set				
Autoencoder-derived pattern analysis	0.89 (0.75–0.97)	NA	95.2	76.5
rCBV	0.92 (0.88–0.98)	3.0	90.0	86.7
**Differentiation of glioblastoma from metastasis**				
Internal validation set				
Autoencoder-derived pattern analysis	0.95 (0.83–0.99)	NA	90.0	85.7
rCBV	0.81 (0.65–0.92)	3.2	70.0	85.7
**Differentiation of metastasis from PCNSL**				
Internal validation set				
Autoencoder-derived pattern analysis	0.90 (0.75–0.98)	NA	81.8	90.0
rCBV	0.59 (0.41–0.76)	3.5	45.4	80.0

*rCBV* relative cerebral blood volume, *PCNSL* primary central nervous system lymphoma.

When distinguishing metastasis from glioblastoma, the CNN had an AUC of 0.89 (95% CI 0.83–0.94), sensitivity of 81.6%, and specificity of 84.4% on the training set. Internal validation gave an AUC of 0.95 (95% CI 0.83–0.99), sensitivity of 90.0%, and specificity of 85.7%. There was no external validation set available for metastasis.

For distinguishing metastasis from PCNSL, the CNN had an AUC of 0.80 (95% CI 0.71–0.87), sensitivity of 74.1%, and specificity of 81.6% on the training set. Internal validation gave an AUC of 0.90 (95% CI 0.75–0.98), sensitivity of 81.8%, and specificity of 90.0%. There was no external validation set available for metastasis.

The assignments of each tissue type in the external validation set are shown in Supplementary Table S1. The assignment of tissue types was similar to that of normally appearing brain tissue located in cluster 4 (44.0%), whereas the tumoral tissues were located in cluster 5 (49.5%) and cluster 3 (29.3%). In the external validation set, the accuracy of differentiating CEL tumors from other tissue types according to cluster frequencies was 86.7%.

### Comparison to conventional rCBV approach

Clustering analysis based on autoencoder-derived temporal patterns had significantly better diagnostic performance when distinguishing PCNSL from glioblastoma than the rCBV approach (AUC 0.79, 95% CI 0.65–0.90) (Delong’s test, *P* < 0.001). In the external validation set, cluster analysis and rCBV had similar diagnostic performance (AUC 0.92, 95% CI 0.88–0.98) (*P* = 0.56) (Table 3).

The clustering analysis of autoencoder-derived temporal patterns also had significantly better diagnostic performance than rCBV for distinguishing metastasis from glioblastoma (AUC 0.81, 95% CI 0.65–0.92; Delong’s test, *P* < 0.001) and metastasis from PCNSL (AUC 0.59, 95% CI 0.41–0.76; Delong’s test, *P* < 0.001).

## Discussion

In this study, a clustering analysis of autoencoder-derived temporal pattern that used information from complex time-signal intensity curves in the whole brain highlighted how the spatial heterogeneity of DSC imaging can be used for tissue characterization. The high-dimensional information included in DSC MRI obtained from all voxels and patients was reduced into nine representative clusters of temporal patterns. These patterns were functionally clustered to separate tissue types and were 91% accurate when used to differentiate between tumor and non-tumor. The differentiation of glioblastoma from metastasis, which was difficult to be achieved using the scalar-based parameters of rCBV methods, was improved when it was based on the dominant clusters of temporal patterns. These dominant clusters offered high diagnostic performance during tumor diagnosis. Furthermore, this autoencoder method does not require model-based calculation of the DSC signal and external validation. The autoencoder effectively learned the complex DSC time-signal intensity curves, and compared with other perfusion methods, provided additional information on intratumoral perfusion heterogeneity and generalizability across centers, which has clinical significance in perfusion analysis.

The autoencoder-derived temporal pattern enabled the use of comprehensive information in the signal-intensity time curves of DSC MRI, including baseline signal intensity, slope and depth of signal decrease, and percentage recovery. Leakage-corrected rCBV reflects an early phase of information by using signal drop and recovery^7^, while percentage signal recovery^23^, derived from the ΔR2* curves obtained from DSC perfusion imaging, reflects a delayed phase of information. These information snapshots, as well as one-dimensional parameters, are affected by the complex interplay of technical (e.g., pulse sequence, contrast agent dose) and pathologic (e.g., vascular permeability, tumor cell volume fraction) factors^8, 13^. For example, the accuracy of rCBV depends on acquisition parameters such as TE and flip angle^14^. The calculation of leakage correction is software dependent^24, 25^, and the percentage signal recovery is also influenced by contrast agent preload and pulse sequence parameters^8^. Indeed, when we compared the average rCBV values, the rCBVs of glioblastoma were 5.52 ± 2.33 (mean ± standard deviation) vs. 7.34 ± 1.51 in the internal and external validation sets, respectively. The average rCBV of PCNSL was 3.77 ± 1.52 vs. 3.38 ± 1.34 in the internal and external validation sets, respectively. The high diagnostic performance of rCBV for differentiating between glioblastoma and PCNSL comes from the higher rCBV values in glioblastoma. The higher rCBV values may be a result of the shorter TE (30 ms) in the external validation set compared with the TE of 40 ms, which would increase the baseline signal and induce a large signal drop^26^. These factors influencing the model-based assumptions are a barrier to the general use of DSC MRI. The autoencoder-derived temporal pattern used in this study did not require complex model-based assumptions, which might lead to broader use of the technique. Also, the method did not need correction based on center or scanner type, which illustrates its potential for use in multicenter DSC MRI temporal pattern analysis.

This method provides an effective way of learning from a large amount of DSC MRI data. Because a single DSC perfusion image can have approximately 3.2 × 10^5^ time-signal intensity curves, achieving supervised learning in all voxels is challenging. Previous studies utilizing DSC perfusion imaging relied on either averaged time-signal intensity curves within a given ROI^27^ or manually selected single or several voxels in the tissue of interest^28, 29^. However, these methods do not accurately represent tumor heterogeneity and require discarding spatial information included in DSC time-signal intensity data. The autoencoder-derived temporal pattern method used entire labeled tissues with a probability matching template, and the spatial mapping of autoencoder features captured relevant spatial information in the clustering method. Spatial heterogeneity is a hallmark of cancer^30^. The visualization of clusters revealed such differences in tumors, e.g., glioblastoma exhibited more intratumoral heterogeneity than PCNSL. Quantifying heterogeneity in representative time-signal intensity patterns will be useful to the investigation of tumor biology.

The autoencoder learned temporal patterns that were subsequently used for supervised learning of tumor diagnosis. In the differentiation of glioblastoma from PCNSL, our method showed high diagnostic performance, with AUCs of 0.93 and 0.89 in the internal and external validation sets, respectively. Also, for distinguishing metastasis from glioblastoma, an AUC of 0.95 was shown in the internal validation. For differentiating metastasis from PCNSL, our method showed an AUC of 0.90 in the internal validation. Machine learning using quantified maps from diffusion-weighted or perfusion-weighted imaging has been applied to the differentiation of the above three diseases^31, 32^, but our autoencoder approach has strengths in that it captures intratumoral heterogeneity by utilizing time-signal intensity curves. Furthermore, the diagnostic performance was high in our method compared with a maximum accuracy of 69.2%^31^ amongst three diseases. Glioblastoma and metastasis can be distinguished from PCNSL based on high rCBV and low percentage of signal recovery relative to the low rCBV and high percentage signal recovery seen in PCNSL^33^. However, neither of these parameters are helpful distinguishing metastasis from glioblastoma, which remains inarguably one of the most difficult distinctions in clinical practice. Indeed, metastatic tumors showed distinct differences from glioblastoma and PCNSL in the dominant cluster, which had a very low relative signal recovery. Furthermore, PCNSL showed the most homogeneous cluster distributions of temporal patterns, whereas metastasis showed vastly different clusters according to the varied cell types. Differentiating the primary tumors of PCNSL and glioblastoma from the secondary brain tumors of metastasis is an important task in neuro-oncology because treatment options differ vastly^34, 35^. The performance of the autoencoder might benefit tumoral diagnoses based on differences in temporal patterns.

This study has several limitations. First, the diagnostic performance of the CNN dropped in the external validation, possibly as a result of overfitting. Although the external validation used a similar DSC acquisition based on the consensus recommendation^3^ and standardized assessment of rCBV^2^, the time-signal intensity curves can nonetheless be affected by technical differences. Thus, the curves might need to be further adjusted if the contrast agent preload and image acquisition protocols are very different. Second, there was no external validation for metastasis because DSC MRI is not commonly performed on patients with suspected brain metastases. Finally, tumors exhibited spatial heterogeneity although dominant clusters were selected to represent tumors. Further sophisticated method to explain entire spatial heterogeneity of DSC time-signal intensity will be needed in the future.

In conclusion, the clustering of autoencoder-derived temporal patterns provided information that was not otherwise accessible with rCBV perfusion methods and showed generalizability across different centers.

## Supplementary Information

Supplementary Information

## Data Availability

The datasets generated during and/or analyzed during the current study are available from the corresponding author on reasonable request.
