# Supplementary material for: Deep-learned time-signal intensity pattern analysis using an autoencoder captures magnetic resonance perfusion heterogeneity for brain tumor differentiation
Source: Sci Rep. 2020 Dec 8;10:21485. doi: 10.1038/s41598-020-78485-x (PMC7723041; doi:10.1038/s41598-020-78485-x)

**Supplemental Materials for**

**Deep-learned Time-Signal intensity Pattern Analysis using Autoencoder Captures Magnetic Resonance Perfusion Heterogeneity for Brain Tumor Differentiation**

Ji Eun Park^1^, Ho Sung Kim^1^, Junkyu Lee^2^, E-Nae Cheong^2^, Ilah Shin^3^, Sung Soo Ahn^3^, Woo Hyun Shim^1,2^

^1^ Department of Radiology and Research Institute of Radiology, University of Ulsan College of Medicine, Asan Medical Center, Seoul 05505, Korea

^2^ Department of Medical Science and Asan Medical Institute of Convergence Science and Technology, Asan Medical Center, University of Ulsan College of Medicine, Seoul, Republic of Korea

^3^ Department of Radiology, Research Institute of Radiological Science and Center for Clinical Imaging Data Science, Yonsei University, South Korea

**Corresponding author:** Ho Sung Kim, M.D., Ph.D.

Department of Radiology and Research Institute of Radiology, University of Ulsan College of Medicine, Asan Medical Center, 43 Olympic-ro 88, Songpa-Gu, Seoul 05505, Korea

Phone: 82-2-3010-1505

E-mail address: radhskim@gmail.com

**Supplementary Table 1.** Brain tissue characterization based on frequency of patterns in the validation set

|  | **Dominant**  **Pattern** | Pattern 0 | Pattern 1 | Pattern 2 | Pattern 3 | Pattern 4 | Pattern 5 | Pattern 6 | Pattern 7 | Pattern 8 |
| --- | --- | --- | --- | --- | --- | --- | --- | --- | --- | --- |
| CEL in glioblastoma | 5 and 3 | 0.90 | 0 | 2.15 | 20.5 | 1.0 | **21.03** | 0.25 | 0.84 | 0.86 |
| CEL in PCNSL | 5 and 2 | 0.93 | 0.1 | 10.05 | 9.4 | 1.21 | **27.50** | 0.33 | 1.00 | 0.72 |
| Gray matter | 2 and 4 | 1.98 | 0 | **41.04** | 0 | 39.2 | 0.28 | 1.77 | 15.61 | 0.11 |
| White matter | 4 and 2 | 0.02 | 0 | 17.81 | 0 | **60.5** | 0 | 2.4 | 19.25 | 0.02 |
| CSF | 8 and 2 | 1.38 | 0 | 21.34 | 0 | 32.2 | **4.51** | 6.29 | 9.54 | **24.71** |

Note. The numbers in the cells are percentages. Necrosis is defined in glioblastoma and metastasis. CEL: contrast-enhancing lesion. PCNSL: primary central nervous system lymphoma.

**Supplementary Figure 1**. Structure of the autoencoder. The encoder is a one-dimensional convolutional layer and the decoder is a two fully-connected layers of neural network. The number of latent spaces was set at five. Note- Conv=convolutional layer; ReLu = rectified linear unit


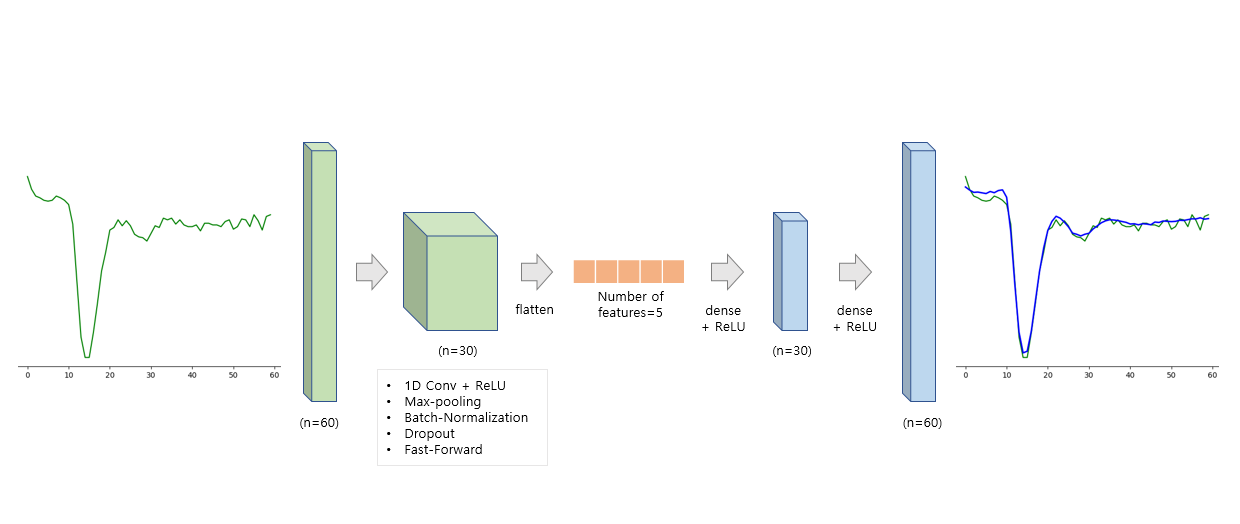


**Supplementary Figure 2**. Structure of the convolutional neural network (CNN) for tumor classification. The CNN was designed with three convolutional blocks, two fully-connected layers, and a softmax layer. The input data size was a 60-element input vector representing the 60 time points of the time-signal intensity curve. Note- Conv=convolutional layer; ReLu = rectified linear unit; PCNSL = primary central nervous system lymphoma


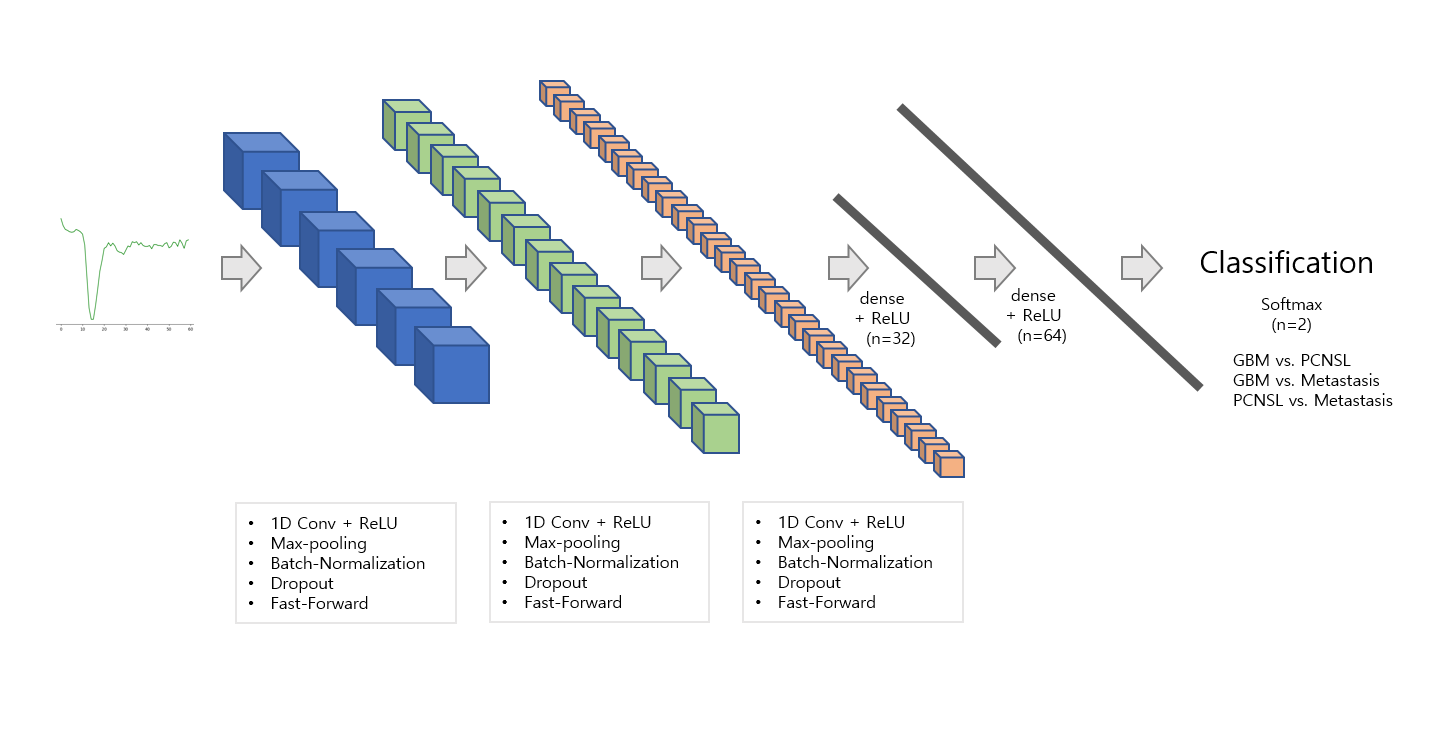

Supplement: Supplementary file 1 — Supplementary Information [file 41598_2020_78485_MOESM1_ESM.docx]
